# Health literacy, health status and health behavior among students and trainees in nursing: a longitudinal cohort study

**DOI:** 10.3389/fmed.2026.1860353

**Published:** 2026-09-30

**Authors:** Ramona Otto, Ivonne-Nadine Jürgensen, Albert Nienhaus, Peter Koch

**Affiliations:** 1 Competence Center for Epidemiology and Health Services Research for Healthcare Professionals (CVcare), Institute for Health Services Research in Dermatology and Nursing (IVDP), University Medical Center Hamburg-Eppendorf, Hamburg, Germany; 2 Department of Nursing and Management, Faculty of Business and Social Science, University of Applied Sciences Hamburg, Hamburg, Germany; 3 Department for Occupational Medicine, Hazardous Substances and Health Sciences (AGG), German Social Accident Insurance for the Health and Welfare Services (BGW), Hamburg, Germany

**Keywords:** health behavior, health literacy, health status, nurses, nursing, psychological wellbeing, students, trainees

## Abstract

**Introduction:**

Nursing trainees and students are exposed to considerable physical and psychological demands early in their careers, which may affect their health status and health behavior. Health literacy has been suggested as a potential resource for maintaining health; however, longitudinal evidence in this population remains scarce.

**Methods:**

This longitudinal cohort study aimed to assess health literacy, health status, and health behavior among 128 nursing trainees and 43 nursing students over one year and to examine their development and potential group differences. Data were collected using paper-pencil and online questionnaires between January 2021 and July 2022. Health literacy (HLS-EU-Q16), psychological wellbeing (WHO-5), self-rated health status, BMI, and health behaviors (smoking, alcohol consumption, physical activity, and nutrition) were assessed.

**Results:**

While average health literacy scores remained stable over time, individual-level changes were heterogeneous. BMI increased significantly over time. Self-rated health status deteriorated significantly among trainees. In the total sample, both self-rated health status and psychological wellbeing showed slight, non-significant decreases. Smoking prevalence increased significantly, whereas other health behaviors remained largely unchanged. No significant associations were found between baseline health literacy and health outcomes or health behaviors at follow-up. Differences between trainees and students were generally small, with BMI being the only outcome consistently higher among trainees.

**Conclusion:**

During the first year of training, nursing trainees and students showed a decline in aspects of health status, while health literacy remained stable. No statistically significant associations were detected between health literacy at baseline and health outcomes or behaviors at follow-up. These findings highlight the need for targeted health promotion strategies in early nursing education. Further research is needed to better understand the development of health literacy throughout nursing job training.

## 1 Introduction

The global nursing workforce is facing an unprecedented shortage, as the World Health Organization estimates a deficit of 4.5 million nurses worldwide (1). Part of tackling this nursing crisis should focus on the training and education of young nurses, preparing them for a long and healthy career in this challenging field. There is a range of issues nurses face in their daily life, such as high physical and psychological demands, time pressure, chronic understaffing and shift work (2). These factors can contribute to elevated stress levels and burnout, and also result in higher sick-leave rates of nurses. A recent umbrella review by Getie et al. found that factors of the workplace contributing to burnout in nurses are the predictability of work tasks, limited opportunities for career progression, moral distress and role conflict, high emotional demands, lack of social support, excessive workloads, and understaffing (2). Especially the combination of these factors with a lack of social support, personal coping strategies and family issues worsens the situation of nurses even further (2).

This situation is particularly challenging for trainees and students in nursing as they are just entering and adapting to this demanding occupation, while at the same time many of them experience a challenging phase in life, transitioning from school and potentially leaving the parental home. Their limited experience in the job makes them even more prone to burnout as a result of high workload (2).

In Germany, nursing qualification can be obtained either through vocational training or through an academic nursing degree program at a university of applied sciences. While vocational training primarily takes place in nursing schools with extensive practical placements, nursing degree programs combine academic coursework with clinical training and place greater emphasis on scientific competencies, management, and evidence-based practice. Both pathways qualify graduates as registered nurses but differ in structure, academic depth, and professional focus.

Aiming at high retention rates of nursing trainees and students, it is therefore a worthwhile approach to shed light on their psychological wellbeing and their general subjective health status as well as how different factors might interrelate with these aspects.

A promising resource for health promotion is the concept of health literacy. While there are several definitions on what health literacy comprises, Sörensen et al. are most commonly referred to, since they offer a comprehensive definition based on the literature. According to them, health literacy is an individual's competencies in accessing, understanding, appraising and applying health-related information (3). In Germany there have been efforts made to encourage the promotion of health literacy. The National Health Literacy Action Plan for Germany demanded strengthening health literacy as one way to improve and maintain physical and psychological health (4).

Health literacy has been increasingly addressed in research (5) and found to be determined by demographic factors such as gender, social and financial status, migration background, education, age and the presence of chronic diseases (6–8). Health literacy is associated with several aspects of health-related outcomes such as psychological wellbeing (9, 10), self-rated health status (11–13) and health behavior (11). A recent cross-sectional study among students enrolled in health-related degree programs also found a positive association between health literacy and healthy lifestyle awareness (14).

In respect to health-related outcomes, it is also important to look into nurses' health behavior. Previous studies found high prevalence rates of cigarette consumption of more than 40% in nursing trainees and students (15–17). In a qualitative study, nurses reported that workplace-specific factors such as high workload and time pressure influence smoking behavior and that smoking in the workplace setting facilitates social interaction and provides a legitimized way of taking breaks (18). Furthermore, alcohol consumption and the lack of physical activity are prevalent among nurses (19, 20) and can contribute to adverse health outcomes.

Based on the literature, the following hypotheses were generated and guided the conduct of the study:

1. Health literacy of nursing trainees and students increases in the first year of their job training.
2. Subjective health status of nursing trainees and students decreases in the first year of their job training.
3. Health literacy of nursing trainees and students at baseline predicts health outcomes at follow-up.
4. The development of health literacy and health outcomes over the first year of training differs between nursing trainees and students.

The purpose of collecting data from nursing trainees and students was to evaluate possible long-term differences in health literacy due to the higher degree of scientific competencies they acquire on their academic educational pathway.

## 2 Methods

### 2.1 Sample and data collection

The longitudinal study was performed between January 2021 and July 2022 in the Northern German federal states of Hamburg, Lower Saxony, Schleswig-Holstein, Mecklenburg-Western Pomerania and Berlin. Initially, trainees were recruited in vocational schools offering generalized nursing education. Eligible vocational schools were identified by online research and contacted regarding their willingness to participate in the study. Using a paper-pencil approach, trainees in their first few weeks of vocational training were invited to participate in the study. For the follow-up, trainees who participated in the baseline survey, were approached via postal invitation one year later.

Nursing students were initially recruited at the Hamburg University of Applied Sciences (*N*ursing & Management Department). They were approached during lectures and asked to fill in online questionnaires at baseline and one year later (follow-up). Each time, students completed the questionnaire during the lecture with the researchers present to answer any questions.

For trainees, cooperating vocational schools disseminated the paper-pencil questionnaires at baseline. As a result, the exact conditions of the recruitment process remain unclear to the researchers and might differ between vocational schools. Since the number of participating vocational schools was high and they were spread across northern Germany, the recruitment process could not be supervised and executed as close as it was the case for the participating University.

No *a priori* sample size or power calculation was performed, as the aim was to approach all eligible vocational schools in northern Germany. However, the final sample size depended on the participation of the schools and trainees.

A total of 2,641 trainees were invited to participate in the baseline survey, of which 556 returned the questionnaire to the study center (response rate 21%). At the midpoint of their vocational training (follow-up), a printed questionnaire was sent to the private addresses of 484 trainees who had given their approval for follow-up surveys. A total of 128 follow-up-questionnaires were eligible for analysis (follow-up rate 26%).

Additionally, 109 students were invited to participate in the baseline survey, of whom 85 filled in the questionnaire (response rate 78%). One year later, these 109 students were invited for the follow-up survey, resulting in 44 filled in questionnaires at follow-up (follow-up rate 40%). We were able to match 43 baseline and follow-up datasets from students to include them into the analyses. The recruitment and follow-up process, including the numbers of participants at each stage, is illustrated in Figure 1.

**Figure 1:**
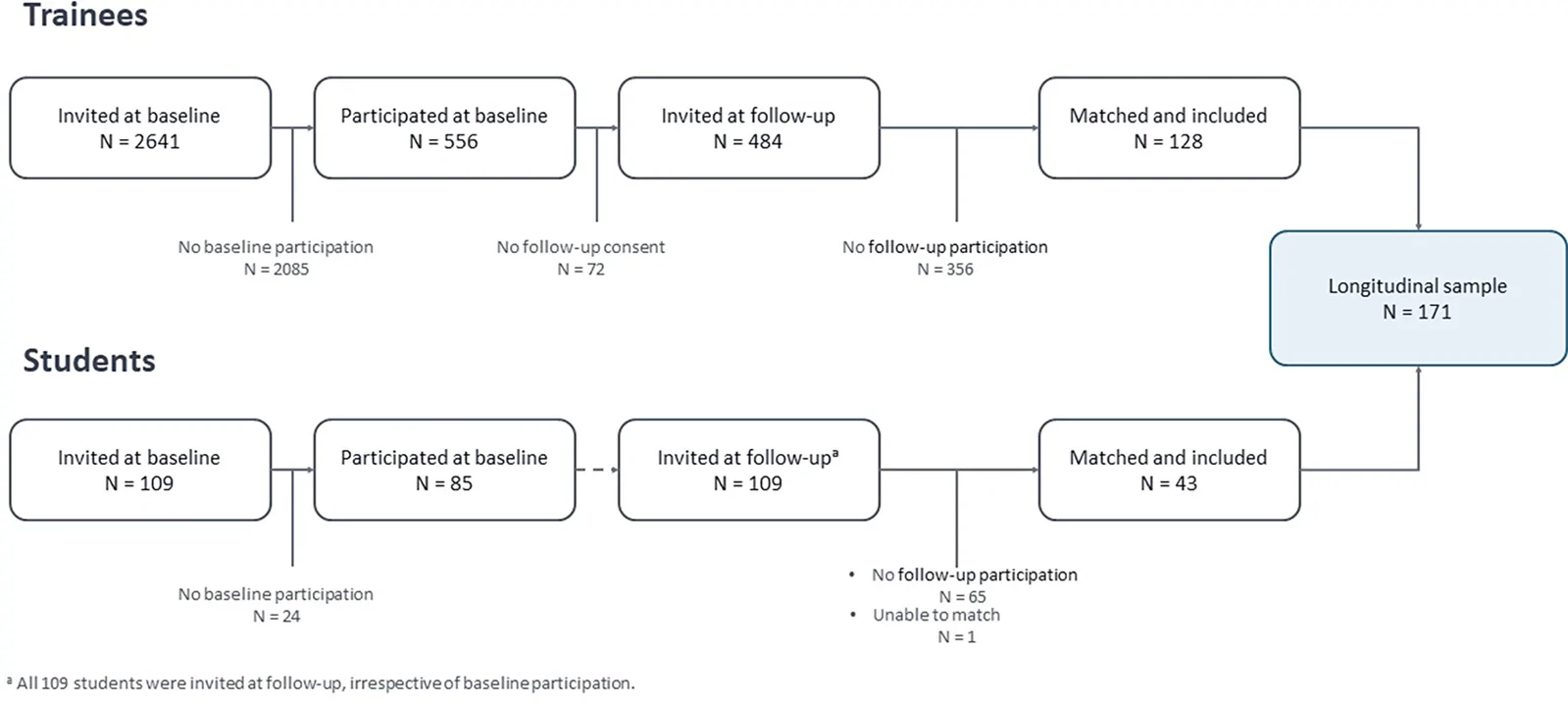
Recruitment and follow-up process of nursing trainees and students.

A detailed report of the baseline results has been published previously (21).

### 2.2 Ethical considerations

An ethics endorsement was obtained from the Hamburg Medical Association (PV5670). Furthermore, the study was approved by the school authorities in the federal states of Berlin, Lower Saxony, Schleswig-Holstein and Mecklenburg-Western Pomerania. The school authority in the federal state of Hamburg did not approve the study, leading to the exclusion of vocational schools located in Hamburg. Study participants were provided with details about the purpose of this study, the fact that participation was voluntary, their right to refuse to answer or withdraw from the study, and the risks and benefits of participation. All participants signed the declaration of consent.

### 2.3 Measures

#### 2.3.1 Demographic information

The baseline questionnaire incorporated several questions on sociodemographic data, covering age, gender, nationality, country of birth and highest level of education.

#### 2.3.2 Health literacy

The short form of the German Health Literacy Survey Questionnaire (HLS-EU-Q16) (22, 23) was used to assess the self-reported health literacy of the cohort. The instrument consists of 16 items scored on a 4-point Likert scale ranging from 1 (very easy) to 4 (very difficult). The instrument touches on three main areas of health literacy: healthcare, disease prevention and health promotion. Following the recommended procedure (23), responses were dichotomized, with “very easy” and “fairly easy” scored as 1 and “fairly hard” and “very hard” scored as 0, and simple sum scores were calculated to provide a score ranging from 0 to 16. Higher scores indicate better health literacy. Based on the sum score, three health literacy levels can be derived: sufficient (13–16 points), problematic (9–12 points) and inadequate (0–8 points). Only participants with at least 14 valid answers were included in further analyses (23). The psychometric properties of the HLS-EU-Q16 are acceptable and its sum score shows a high correlation with the score of the long version (23). Cronbach's alpha coefficient for the HLS-EU-Q16 was 0.85 at baseline and 0.89 at follow-up in the present study, indicating good internal consistency. Health literacy was assessed both at baseline and follow-up.

#### 2.3.3 Health status

Three indicators of health status were measured:

*Self-rated health status* was assessed subjectively using a 5-point Likert scale (1 = poor, 2 = less good, 3 = good, 4 = very good, 5 = excellent) (24). Categories were dichotomized between “poor” (less good/poor) and “good” (excellent/very good/good).
*Psychological wellbeing* over the previous two weeks was measured using the German version of the 5-item World Health Organization Wellbeing Index (WHO-5) (25). The instrument contains 5 questions with response options ranging from 0 (at no time) to 5 (all of the time). Item scores were summed up to provide a total score between 0 and 25, with higher scores indicating better psychological wellbeing. The index was dichotomized using the cutoff value suggested in the literature (25); consequently, scores under 13 points were categorized as poor psychological wellbeing. Cronbach's alpha coefficient for the WHO-5 was 0.88 at baseline and 0.89 at follow-up in the present study, indicating good internal consistency.
*Body Mass Index (BMI)* was calculated based on self-reported body height and weight. BMI was categorized as follows: underweight (BMI < 18.5), normal weight (BMI 18.5-<25), overweight (BMI 25-< 30) and obese (BMI ≥ 30) (26).

#### 2.3.4 Health behavior

Four indicators measuring health behavior were measured as follows:

*Smoking behavior* was measured by one item, asking whether participants were currently smoking. The possible responses were “Yes, daily”, “Yes, occasionally”, “No, I have smoked in the past” and “No, I have never smoked”. Responses were summarized as “yes” (daily or occasionally) and “no” (non-smoker or ex-smoker) (27).
*Alcohol consumption* was assessed using the AUDIT-C questionnaire (28), consisting of three questions. Item scores were summed up to provide a total score ranging from 0 to 12 points, with higher scores indicating higher levels of alcohol consumption. The index was dichotomized using different cutoff values for risky alcohol consumption for men and women (men: > 4 points; women: >3 points).
*Physical activity* was assessed by one question capturing how frequently each participant is physically active. The possible responses were “no physical activity”, “less than 1 h per week”, “regularly, 1-<2 h per week”, “regularly, 2–4 h per week” and “regularly, more than 4 h per week” (29). Responses were dichotomized between “sufficient” (2–4 h per week or >4 h per week) and “insufficient” (1-<2 h per week, < 1 h per week or none) activity levels.
*Eating behavior* was measured using self-reports based on a food frequency list including 15 food categories (30). Responses were analyzed in accordance with the authors' framework to provide a score ranging from 0 to 30. Based on their score, participants were assigned to the following categories: optimal nutritional pattern (16–30 points), normal nutritional pattern (13–15 points) and unfavorable nutritional pattern (0–12 points). A binary variable was computed by summarizing optimal and normal nutritional patterns into one category. Fast food consumption was analyzed separately and dichotomized between “≤ once/week” and “> once/week”.

### 2.4 Data analyses

Descriptive statistics were applied to characterize the cohort across all relevant variables. Multivariable regression analyses were conducted to examine whether health literacy at baseline was associated with psychological wellbeing, self-rated health, BMI, alcohol consumption, physical activity, smoking status, and nutrition pattern at follow-up. Multiple linear regression was used for the outcomes psychological wellbeing, self-rated health, BMI, alcohol consumption, physical activity, and nutrition pattern., whereas binary logistic regression was used for smoking status. Additional linear regression models examined the associations of baseline health behaviors (alcohol consumption, physical activity, smoking, and nutrition pattern), with self-rated health and psychological wellbeing at follow-up. All models were adjusted for the respective baseline outcome, age, and gender. Linear regression assumptions were assessed using residual and P–P plots, the Durbin–Watson statistic, variance inflation factors, standardized residuals, Mahalanobis distance, and Cook's distance. Model fit of the binary logistic regression was assessed using the Hosmer–Lemeshow test. For the BMI model, a sensitivity analysis excluding four observations with unusually large but verified BMI changes was performed.

Dropout analyses were performed by comparing baseline characteristics between responders and non-responders using chi-square tests and analysis of variance (ANOVA). All statistical analyses were performed using IBM SPSS for Windows Version 27. Results with a *p*-value under the threshold of 0.05 were considered statistically significant. Given the exploratory nature of the study, no *p*-value correction for multiple testing was applied. Missing data were handled using listwise deletion in the multivariable regression analyses. Gender-diverse participants (*N* = 1) were randomly assigned to one of the binary gender groups for the purpose of the analyses as the gender-diverse group was too small to be analyzed separately. Sensitivity analyses were conducted in which gender was treated as missing for the participant identifying as gender-diverse.

Groups of nominal data were compared with Pearson's chi-squared test. The t test was used for normally distributed data. In order to examine changes in dichotomous indicators of health behavior, the McNemar test was calculated. The time courses of health literacy, self-rated health status, psychological wellbeing and BMI were examined using linear mixed-effects models. Measurement occasion, educational pathway and the interaction between measurement occasion and educational pathway were included as fixed effects while accounting for repeated measurements using a participant-level random intercept. Models were estimated using restricted maximum likelihood, with estimated marginal means used to assess changes over time within each educational pathway.

## 3 Results

### 3.1 Characteristics of the sample

There were 171 participants included in the follow-up analysis. The group consisted of 128 trainees and 43 students. The mean age at baseline was 25 years (SD = 8.1) ranging from 17 to 57. The majority of the sample was female (80%) and had German nationality (88%). More detailed information is displayed in Table 1. Sensitivity analyses in which gender was treated as missing for the gender-diverse participant did not alter the statistical significance or interpretation of any findings.

**Table 1:** Demographic characteristics of the study cohort.

Demographic variable	Category	Female (*N* = 136)	Male (*N* = 35)	Total (*N* = 171)	*p*
Age	X (SD)	25.5 (9.4)	25.2 (8.1)	25.5 (9.1)	0.869^a^
	Range	17–62	18–57	17–62	
Nationality	German	122 (90%)	28 (80%)	150 (88%)	0.119^b^
	other	14 (10%)	7 (20%)	21 (12%)	
	n missing	0	0	0	
School education	Lower secondary school (Hauptschule)	7 (5%)	3 (9%)	10 (6%)	0.032^b^
	Higher secondary school (Realschule)	47 (35%)	8 (23%)	55 (33%)	
	Vocational training college (Fachhochschule)	19 (14%)	3 (9%)	22 (13%)	
	A-Levels (Abitur)	61 (46%)	19 (54%)	80 (47%)	
	Other	0 (0%)	2 (6%)	2 (1%)	
	n missing	2	0	2	
Federal state	Hamburg	35 (26%)	8 (23%)	43 (25%)	0.878^b^
	Lower Saxony	66 (49%)	18 (51%)	84 (49%)	
	Schleswig-Holstein	13 (10%)	5 (14%)	18 (11%)	
	Mecklenburg-Western Pomerania	7 (5%)	1 (3%)	8 (5%)	
	Berlin	15 (11%)	3 (9%)	18 (11%)	
Education path	Trainee	101 (74%)	27 (77%)	128 (75%)	0.726^b^
	Student	35 (26%)	8 (23%)	43 (25%)	

a ANOVA.

b Pearson's chi-squared test.

### 3.2 Drop-out analyses

Dropout analyses indicated some differences between responders and non-responders. Among trainees, smoking prevalence was significantly higher among non-responders (*p* < 0.001), whereas the level of school education was significantly higher among responders (*p* = 0.005). Among students, BMI (*p* = 0.028) and the proportion of participants with non-German nationality (*p* = 0.045) were significantly higher among non-responders. No significant differences were observed for age, gender, alcohol consumption, physical activity, nutrition, health literacy, psychological wellbeing, or self-rated health status in either group (all *p* > 0.05).

### 3.3 Health literacy (HL)

At baseline 55% of the sample expressed adequate health literacy while at follow-up this number went up to 63%. This change was not statistically significant [χ²(167) = 1.818, *p* = 0.178]. Average HL score was 12.6 (SD = 2.684) at baseline and 12.9 (SD = 2.919) at follow-up. The linear mixed-effects model showed no significant change in HL scores over time (*p* = 0.224) and no interaction between time and educational pathway (*p* = 0.749). At both timepoints men had higher HL scores than women (Baseline: 13.0 (SD = 2.774) vs. 12.6 (SD = 2.663); Follow-up: 13.4 (SD = 2.216) vs. 12.8 (SD = 3.069), these differences were not statistically significant (Baseline: *p* = 0.385; Follow-Up: *p* = 0.312).

The development of health literacy over time was heterogeneous within the sample. Figure 2 shows the distribution of the sample displaying HL scores at baseline and follow-up. 38% of the sample decreased their HL level, 17% maintained it and 43% improved it. There were no statistically significant associations between the change in HL scores and age [F(1, 165) = 0.135, *p* = 0.713], gender [χ²(2) = 0.587, *p* = 0.746], or nationality [χ²(2) = 2.603, *p* = 0.272]. Based on these results, hypothesis 1 is rejected.

**Figure 2:**
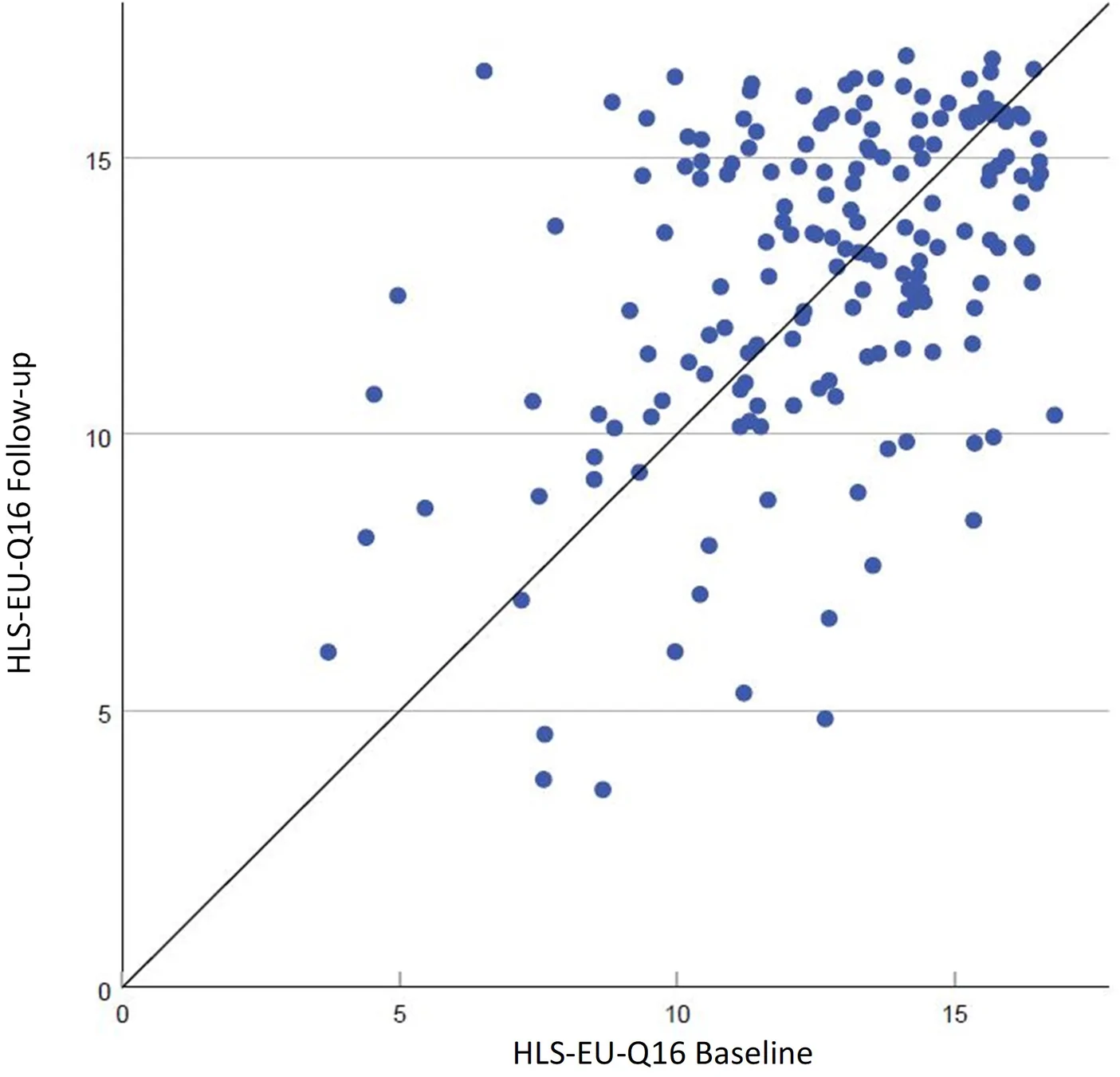
Health literacy scores at baseline and follow-up. The diagonal line indicates no change; points above the line represent an increase and points below the line represent a decrease in health literacy scores.

### 3.4 Health status, BMI and psychological wellbeing

At baseline, 43% of the cohort rated their health status as excellent or very good, 46% said it was good and 11% expressed a less good health status. At follow-up, only 33% had an excellent or very good health status 49% reported good health status and 17% rated their health status as less good or bad. The linear mixed-effects model showed no statistically significant overall change in self-rated health status over time (*p* = 0.069) and no interaction between time and educational pathway (*p* = 0.131). Self-rated health status decreased significantly among trainees (*p* = 0.001), while it remained stable among students (*p* = 0.856). Based on these results, hypothesis 2 cannot be confirmed for the whole sample.

Among the whole sample, the linear mixed-effects model showed that BMI increased statistically significantly from an average of 24.7 at baseline to 25.2 at follow-up (*p* = 0.005) and that BMI values were higher among trainees than among students (*p* = 0.002), with no interaction between time and educational pathway (*p* = 0.784). This resulted in an increase of participants classified as overweight or obese from 36% at baseline to 42% at follow-up.

The share of participants expressing poor psychological wellbeing remained stable at 39% at both data points. However, looking at the WHO-5 score, reflecting psychological wellbeing, there was a slight decrease from an average of 13.6 at baseline to 13.0 at follow-up. The linear mixed-effects model showed no statistically significant change over time (*p* = 0.569) and no interaction between time and educational pathway (*p* = 0.241), although WHO-5 scores were significantly higher among trainees across both time points (*p* = 0.019).

Women expressed lower average WHO-5 scores at baseline and at follow-up compared to men, however this difference was only statistically significant at baseline (*p* = 0.004).

### 3.5 Health behavior

The proportion of participants meeting at least 2 h of physical activity per week decreased from 65% at baseline to 60% at follow-up (McNemar test, *N* = 168 χ² = 1.500, *p* = 0.220).

At baseline 44% of the sample showed unfavorable nutrition pattern, while 56% had normal or favorable nutrition. At follow-up, the proportion of unfavorable nutrition decreased to 39%. This change was not statistically significant [t(161) = 0.446, *p* = 0.656].

There was a statistically significant increase in smoking prevalence from 30% at baseline to 35% at follow-up (McNemar test, *N* = 167, χ² = 5.82, *p* = 0.016).

The proportion of trainees and students showing risky alcohol consumption increased by 6% within the first year of job training (29% at baseline vs. 35% at follow-up), however paired t-test showed no statistical significance (McNemar test, *N* = 169, χ² = 2.531, *p* = 0.112).

### 3.6 Associations of health literacy and health status

There was no statistically significant association between HL at baseline and psychological wellbeing (*p* = 0.257), self-reported health status (*p* = 0.477) or BMI (*p* = 0.492) at follow-up. The results of the multivariable regression analyses are presented in Table 2. Sensitivity analysis excluding four observations with unusually large BMI changes did not materially alter the findings. Based on these results, hypothesis 3 is rejected.

**Table 2:** Multivariate analyses of associations between health literacy and different aspects of health status.

	Health Literacy			
Outcome at Follow-Up^a^	Beta (standardized)	B (unstandardized)	95% CI for B	*p*
Psychological Well-Being (WHO-5)	0.082	0.170	−0.13–0.47	0.257
Self-Rated Health Status	−0.054	−0.016	−0.06–0.03	0.477
BMI	0.021	0.044	−0.08–0.17	0.492

a Adjusted for age, gender and outcome variable at baseline at each analysis.

### 3.7 Associations of health literacy and health behavior

There was no statistically significant association between HL at baseline and alcohol consumption (*p* = 0.634), physical activity (*p* = 0.313), smoking (*p* = 0.211) or nutrition pattern (*p* = 0.797) at follow-up.

### 3.8 Associations of health behavior and health status

Multivariate analysis showed no statistically significant associations of alcohol consumption at baseline (*p* = 0.768), smoking at baseline (*p* = 0.239) or nutrition pattern at baseline (*p* = 0.952) on self-rated health status at follow up. Higher levels of physical activity at baseline were associated with higher self-rated health status at follow-up (*p* = 0.008).

Multivariate analysis showed no statistically significant association of alcohol consumption at baseline (*p* = 0.621) or smoking at baseline (*p* = 0.076) or nutrition pattern at baseline (*p* = 0.627) on self-rated health status at follow up. Favorable nutrition pattern at baseline was associated with higher psychological wellbeing (*p* = 0.022). Higher levels of physical activity at baseline were associated with higher psychological wellbeing at follow-up (*p* = 0.052), however this *p*-value was just above the significance level.

### 3.9 Differences between trainees and students

Table 3 shows that health literacy remained stable in both groups during the observational time with no significant difference between time points. Furthermore, health literacy did not differ statistically significantly between trainees and students at baseline [estimated mean difference = −0.20, 95% CI (−1.17, 0.78), *p* = 0.693] or at follow-up [estimated mean difference = −0.36, 95% CI (−1.34, 0.62), *p* = 0.469]. The linear mixed-effects model showed no interaction between time and educational pathway (*p* = 0.749). Students' psychological wellbeing at baseline was significantly lower than in trainees [11.7 vs. 14.2; estimated mean difference = 2.48, 95% CI (0.62, 4.33), *p* = 0.009]. At follow-up, the difference between trainees and students was not statistically significant [estimated mean difference = 1.30, 95% CI (−0.56, 3.16), *p* = 0.170]. In the time course, trainees' psychological wellbeing declined while students show slight improvements, however both changes over time are not statistically significant. The linear mixed-effects model showed no significant overall change over time (*p* = 0.569) or interaction between time and educational pathway (*p* = 0.241), while WHO-5 scores were significantly higher among trainees across both time points (*p* = 0.019). Self-rated health status of trainees was statistically significantly higher than in students at baseline [3.5 vs. 3.2; estimated mean difference = 0.28, 95% CI (0.01, 0.56), *p* = 0.044]. At follow-up, the difference between trainees and students was not statistically significant [estimated mean difference = 0.06, 95% CI (−0.22, 0.33), *p* = 0.674]. However, during their first year of job training, trainees self-rated health status declined significantly, while students' values remained stable. The interaction between time and educational pathway was not statistically significant (*p* = 0.131), indicating that there was no statistically significant difference in the longitudinal changes between trainees and students. BMI was significantly higher among trainees at baseline [25.5 vs. 22.5; estimated mean difference = 3.00, 95% CI (1.16, 4.84), *p* = 0.002] and follow-up [26.0 vs. 23.1; estimated mean difference = 2.89, 95% CI (1.05, 4.73), *p* = 0.002] compared to students. The linear mixed-effects model showed a significant increase in BMI over time (*p* = 0.005), while the interaction between time and educational pathway was not statistically significant (*p* = 0.784). As none of the interactions between time and educational pathway were statistically significant, hypothesis 4 was not supported.

**Table 3:** Changes in main outcomes from baseline to follow-up by educational pathway.

Outcome variable	Category	Baseline Mean (SD)	Follow-up Mean (SD)	Estimated mean change	95% CI	p	Cohen's d
Health Literacy (HLS-EU-Q16) 0–16	Trainee (*N* = 124)	12.6 (2.8)	12.8 (3.0)	0.23	−0.28–0.74	0.375	0.07
	Student (*N* = 43)	12.8 (2.3)	13.2 (2.8)	0.40	−0.48–1.27	0.373	0.14
	Total (*N* = 167)	12.7 (2.7)	12.9 (2.9)	0.31	−0.19–0.82	0.224	0.09
Psychological Well-Being (WHO-5) 0–25	Trainee (*N* = 123)	14.1 (5.5)	13.3 (5.4)	−0.87	−1.87–0.13	0.087	−0.16
	Student (*N* = 43)	11.7 (4.7)	12.0 (5.7)	0.30	−1.40–2.00	0.726	0.04
	Total (*N* = 166)	13.5 (5.4)	13.0 (5.5)	−0.29	−1.27–0.70	0.569	−0.10
Self-Rated Health Status 1–5	Trainee (*N* = 125)	3.5 (0.8)	3.2 (0.8)	**−0.25**	**−0.39–−0.10**	**0.001**	**−0.31**
	Student (*N* = 43)	3.2 (0.8)	3.2 (0.8)	−0.02	−0.27–0.23	0.856	−0.02
	Total (*N* = 168)	3.4 (0.8)	3.2 (0.8)	−0.14	−0.28–0.01	0.069	−0.22
BMI	Trainee (*N* = 121)	25.5 (5.7)	26.0 (5.9)	**0.50**	**0.11–0.88**	**0.012**	**0.26**
	Student (*N* = 43)	22.5 (3.2)	23.1 (3.6)	0.60	−0.05–1.25	0.069	0.22
	Total (*N* = 164)	24.7 (5.3)	25.2 (5.5)	**0.55**	**0.17–0.93**	**0.005**	**0.24**

Estimated mean changes, 95% confidence intervals, and *p*-values were derived from the linear mixed-effects models and represent follow-up minus baseline. Total estimates represent model-based marginal means across educational pathways.

Cohen's d was calculated as the mean individual change divided by the standard deviation of the individual changes.

Bold values indicate statistically significant changes (p < 0.05).

## 4 Discussion

The present study followed a cohort of 171 nursing trainees and students over the course of one year. This study aimed to assess health literacy, health status, and health behavior among students and trainees and to investigate longitudinal developments as well as group differences.

### 4.1 Health status and psychological wellbeing

Most participants rated their health status good to excellent at both timepoints, while trainees reported a decline over time. The observed prevalence is in line with a study performed in Hungary that found 44% of nurses with a good or excellent self-rated health status (31) and a study from Poland showing that 12% of participating nurses were dissatisfied with their health (32).

Psychological wellbeing remained rather stable between the two timepoints. It is however important to point out that the proportion of participants expressing poor psychological wellbeing, according to the WHO-5 questionnaire, was quite high at 39% at both timepoints.

Furthermore, BMI increased between baseline and follow-up leading to 42% of the cohort being classified as overweight or obese at follow-up.

Taken together, it is vivid, that participating trainees and students expressed an overall decrease in different aspects of their health status within their first year of job training, while psychological wellbeing remained largely stable. In consideration of research pointing at the numerous physical strains of nursing workplaces this development seems to be understandable. A systematic review by Narbona-Gálvez et al. identified time management, workload, adaptation to work environment, pressure, lack of experience, under-resourced work environments and other demands to put novice nurses under a lot of stress (33).

A scoping review found psychosocial risks associated with healthcare jobs, such as lack of personal protective equipment, work organization, moral injury, social and societal pressure and new policies and procedures following the covid19 pandemic to impact healthcare worker's overall wellbeing (34). Also, high rates of burnout were found among nurses negatively impacting their quality of life (35).

### 4.2 Health behavior

Among this cohort the proportion of participants meeting at least 2 h of physical activity per week decreased over the course of one year. At the same time, the share of trainees showing unfavorable nutrition pattern decreased. The prevalence of risky alcohol consumption increased within this cohort. All of these changes were not statistically significant. There was however a statistically significant increase in smoking prevalence. Furthermore, these values exceed the pooled prevalence of 27% among nursing students a meta-analysis found in 2020 (36).

Studies show that shift work is associated with different unhealthy behavior patterns like smoking and alcohol consumption (37, 38). The share of participants working in shifts might be higher after one year of job training compared to the very beginning. This might have a negative impact on health behavior. As we did not assess whether participants worked in shifts, we can however not say, whether this played a role in our cohort.

### 4.3 Health literacy

At the beginning of their job training more than half of the participants expressed adequate health literacy according to the HLS-EU-Q16 questionnaire. Health literacy scores remained largely stable over the first year of job training, however more participants reached the threshold of 13 points, resulting in an increased proportion of participants classified as having “adequate” health literacy at follow-up.

There were no significant associations between the change in health literacy and age, gender, nationality and educational pathway. Furthermore, no significant associations could be found looking at health literacy at baseline as a predictor of different aspects of health status or health behavior. These results were surprising and require cautious interpretation.

Looking into the literature, there are numerous studies pointing at associations between health literacy and health related outcomes, such as physical and psychological health status (9–13). In accordance with this research was the analysis of the baseline data of this study, which found significant associations between health literacy and self-rated health status and psychological wellbeing, as well as mediating effects of self-efficacy (21). These effects did however not show in the longitudinal analyses. In general, longitudinal studies on the predicting value of health literacy are scarce. In a prospective cohort study on trainees from various professions, Koch et al. found increased odds ratios of having poor psychological wellbeing for persons with inadequate or problematic health literacy (10). Another longitudinal study on people with type-1 diabetes, however, found inconclusive results concerning the influence of health literacy on different health markers such as blood pressure (39).

Previous cross-sectional studies found correlations between health literacy and health promoting behavior (11, 40), however in this longitudinal analysis, no such relationships could be observed. This is in line with a similar study on trainees, which did not find associations between health literacy at baseline and health behavior at follow-up (10).

This study assessed health literacy using the HLS-EU-Q16 questionnaire, a validated and often used tool. However, there are various other instruments used, some of which focus on specific aspects, such as digital health literacy, nutrition literacy or dental literacy. This circumstance makes it somewhat hard to compare study results. Aside from this, there is a lack of comparable research investigating health literacy in nurses. More research in this field is needed, as the relevance of nurses' health literacy goes beyond the effects it potentially has on their own health. In their profession as nurses, they will be required to pass information on to their patients. Their level of health literacy does consequently not only play a role for themselves but also affects their patients (41).

### 4.4 Differences between students and trainees

Within this cohort, slight differences were observed between students and trainees. At baseline, students reported significantly lower psychological wellbeing than trainees. During the follow-up period, trainees' health status declined significantly while students remained stable. However, the interaction between time and educational pathway was not statistically significant, indicating no evidence of different longitudinal changes between the groups. BMI was higher among trainees at both measurement points. Although BMI increased in both groups over time, this increase was statistically significant only among trainees. However, the interaction between time and educational pathway was not statistically significant. Concerning health literacy, there were no significant group differences at any point. Overall, the observed differences were small. Academic nursing education is still relatively new in Germany, and research focusing on this population remains limited. To our knowledge, no previous studies have compared nursing trainees and nursing students in Germany during their first year of training.

## 5 Limitations

This study has several limitations. The recruitment of vocational trainees at baseline was executed by cooperating vocational schools, who distributed the paper-pencil questionnaires. As a result, the researchers had limited control over the recruitment procedures. There might have been differences between the vocational schools and it could not be ensured that all potential participants were invited to the study. This lack of standardization could have influenced participation rates.

Furthermore, the recruitment and data collection procedures differed between vocational trainees and university nursing students. Trainees completed paper-pencil questionnaires, whereas students completed online questionnaires in a classroom setting with researchers present. While data collection among university students was closely supervised by the research team, this was not feasible for the vocational schools due to their number and geographic dispersion. These differences may have introduced measurement mode bias and limit the comparability of the two groups. Moreover, because the groups were recruited from different institutions, observed group differences cannot be attributed solely to the educational pathway.

The baseline response rate among vocational trainees was relatively low (21%), which might introduce selection bias, including healthy volunteer bias. Trainees who chose to participate may differ systematically from those who did not, for example regarding motivation, health status, or health literacy. Consequently, the generalizability of the findings to all nursing trainees in Germany is limited. The low follow-up rate (26%) between the two time points represents an additional limitation and resulted in substantial attrition. Although follow-up questionnaires were sent only to trainees who had provided consent for further contact, loss to follow-up may have introduced attrition bias. Dropout analyses indicated some systematic differences between responders and non-responders; however, differences in attrition rates regarding smoking behavior and differences in education, BMI, and nationality suggest that a certain degree of bias cannot be excluded. As these characteristics may be associated with health literacy and health behavior, attrition may have resulted in an over- or underestimation of the observed longitudinal changes and associations. The direction and magnitude of this potential bias cannot be determined. Consequently, the findings may not be generalizable to all nursing trainees and students.

All data were based on self-reported questionnaires, which are subject to reporting biases such as social desirability and recall bias. Furthermore, no objective health measures, such as measured height and weight, were collected. Despite these limitations, the longitudinal design and the inclusion of multiple training settings across several federal states represent important strengths of the study.

Furthermore, the study was performed during the COVID-19 pandemic, which potentially influenced participants' perception of their psychological wellbeing and health status. This might have also contributed to low response rates among nurses due to the multiple challenges they were facing. Finally, the imbalanced gender ratio in the investigated cohort is a limitation worth noting. It is, however, reflective of the target population. Nevertheless, the limited follow-up sample and small subgroup sizes, especially the student subgroup (*N* = 43), limited the statistical power of the study, particularly for the detection of associations or differences in changes between the educational pathways. Consequently, some potential differences may have remained undetected.

## 6 Conclusion

This study followed a group of nursing trainees and students throughout their first year of job training, observing health literacy, health behavior and health status. On average, health literacy scores were high and remained stable during the survey period, however participants' individual development was heterogeneous irrespective of the educational pathway. In the total sample, self-rated health status showed a slight, non-significant decline, while psychological wellbeing remained largely stable. No statistically significant associations were detected between health literacy at baseline and health behavior or health status at follow-up. Furthermore, only some aspects of health behavior at baseline were associated with psychological wellbeing at follow-up.

It would be desirable to employ a qualitative design to further investigate and understand the development of health literacy throughout nursing job training.

## Data Availability

The raw data supporting the conclusions of this article will be made available by the authors, without undue reservation.
